# Defining the microbial transcriptional response to colitis through integrated host and microbiome profiling

**DOI:** 10.1038/ismej.2016.40

**Published:** 2016-03-22

**Authors:** Nicholas Edward Ilott, Julia Bollrath, Camille Danne, Chris Schiering, Matthew Shale, Krista Adelmann, Thomas Krausgruber, Andreas Heger, David Sims, Fiona Powrie

**Affiliations:** 1 Kennedy Institute of Rheumatology, University of Oxford, Oxford, UK; 2 Institute for Tumor Biology and Experimental Therapy, Georg-Speyer-Haus, Frankfurt am Main, Germany; 3 The Francis Crick Institute, Mill Hill Laboratory, London, UK; 4 Nuffield Department of Medicine, Translational Gastroenterology Unit, Experimental Medicine, John Radcliffe Hospital, Oxford, UK; 5 CeMM Research Center for Molecular Medicine of the Austrian Academy of Sciences, Vienna, Austria; 6 Department of Physiology, Computational Genomics Analysis and Training (CGAT), MRC Functional Genomics Unit, Anatomy and Genetics, University of Oxford, Oxford, UK

**Keywords:** Metagenomics, Bacteria

## Abstract

The gut microbiome is significantly altered in inflammatory bowel diseases, but the basis of these changes is not well understood. We have combined metagenomic and metatranscriptomic profiling of the gut microbiome to assess modifications to both bacterial community structure and transcriptional activity in a mouse model of colitis. By using transcriptomic analysis of colonic tissue and luminal RNA derived from the host, we have also characterised how host transcription relates to the microbial transcriptional response in inflammation. In colitis, increased abundance and transcription of diverse microbial gene families involved in responses to nutrient deprivation, antimicrobial peptide production and oxidative stress support an adaptation of multiple commensal genera to withstand a diverse set of environmental stressors in the inflammatory environment. These data are supported by a transcriptional signature of activated macrophages and granulocytes in the gut lumen during colitis, a signature that includes the transcription of the key antimicrobial genes *S100a8* and *S100a9* (calprotectin). Genes involved in microbial resistance to oxidative stress, including Dps/ferritin, Fe-dependent peroxidase and glutathione *S*-transferase were identified as changing to a greater extent at the level of transcription than would be predicted by DNA abundance changes, implicating a role for increased oxygen tension and/or host-derived reactive oxygen species in driving transcriptional changes in commensal microbes.

**Supplementary information:**

The online version of this article (doi:10.1038/ismej.2016.40) contains supplementary material, which is available to authorized users.

## Introduction

Inflammatory bowel disease (IBD), which includes ulcerative colitis (UC) and Crohn’s disease, is a chronic and recurring inflammatory condition of the gastrointestinal tract that has been linked to dysregulated mucosal immunity and alterations in gut microbial communities (Ott et al., 2004; Sokol et al., 2008; Frank et al., 2007, 2011; Packey and Sartor, 2009; Erickson et al., 2012; Morgan et al., 2012). High throughput studies of the intestinal microbiota using 16S rRNA sequencing or shotgun metagenomics in IBD patients have consistently shown a state of dysbiosis, which is characterised by a reduction in biodiversity, a reduction in members of the firmicutes phylum (particularly short-chain fatty acid producing Ruminococcaceae, *Odoribacter*, Leuconostocaceae, *Phascolarctobacterium* and *Roseburia*) and an increase in Gammaproteobacteria (Kostic et al., 2014). Furthermore, functional inference using 16S rRNA data has revealed increased abundance of metabolic pathways related to metabolism in the presence of oxidative stress as well as increases in nutrient transport systems and decreased amino acid metabolism (Morgan et al., 2012). Nevertheless, heterogeneity in IBD pathology and aetiology creates a problem for generalising microbial associations across the spectrum of disease.

Mouse models of IBD represent a valuable tool to identify microbial pathways involved in inflammation as host genetics and environmental influences can be easily controlled. However, different models capture different aspects of human disease and thus differential cross-talk between the host and the microbiota. Comprehensive analyses of microbial communities across multiple models of IBD are therefore imperative to shed light on different aspects of immune modulation of the microbiota and vice versa. Community profiling studies have been performed across multiple models of IBD, with taxonomic changes being dependent on the model under investigation (Gkouskou et al., 2014). For example, mice deficient for the transcription factor T-bet (T-bet^−/−^) develop spontaneous colitis in the absence of an adaptive immune system (T-bet^−/−^ Rag2^−/−^ UC (TRUC)), which is driven by dendritic cell-derived tumour necrosis factor alpha and is dependent on the commensal microbiota (Garrett et al., 2007, 2010). In this model, aberrant innate immune control of commensal microbes results in increased abundance of colitogenic bacteria, including an increased abundance of the tetrathionate metabolising Enterobacteriaceae as well as an inferred increase in potentially immunogenic flagellar components (Rooks et al., 2014). In contrast, the dextran sodium sulphate (DSS) model of colitis is driven by direct chemical perturbation of the epithelium, resulting in microbiota-dependent induction of inflammation (Perše and Cerar, 2012). Although DSS colitis has also been linked to increased abundance of multiple families of bacteria, including the Enterobacteriaceae (Okayasu et al., 1990; Schwab et al., 2014), in contrast to the TRUC model, it is associated with a decreased abundance of flagellin transcripts (Berry et al., 2012; Schwab et al., 2014), suggesting differences in the pathogenic mechanisms in these models.

Previous community profiling studies in colitis have been carried out using 16s rRNA gene surveys, shotgun metagenomics or metatranscriptomics. However, to our knowledge there have been no studies that combine community DNA sequencing (metagenomics) with community RNA sequencing (metatranscriptomics), which has limited interpretation of functional changes. To address the gap in our knowledge of metagenome-wide transcriptional responses in colitis, we have combined shotgun metagenomic and metatranscriptomic sequencing in the *Helicobacter hepaticus* (*H. hepaticus*) model of colitis (Kullberg et al., 2006). This model reflects a multi-factorial aetiology relying on a bacterial trigger in combination with an impairment of immune function through blockade of IL10 signalling. It is highly relevant to the aetiopathogenesis of a subset of IBD as loss of function mutations in IL10 receptors (*IL10R1* and *IL10R2*) and *IL10* result in early onset monogenic forms of the disease, manifesting as aggressive forms of IBD in children (Glocker et al., 2009; Kotlarz et al., 2012; Uhlig et al., 2014). Furthermore, significant associations of variants at the *IL10* locus with Crohn’s disease and UC in genome-wide association studies (Franke et al., 2010; Doecke et al., 2013) support the role of IL10 signalling in contributing to polygenic risk of disease. Our findings support data from human IBD studies regarding changes in oxidative stress pathways in the microbiota and extend these findings to include robust associations between colitis and multiple pathways implicated in adaptations of commensals to host defence responses, including antimicrobial peptide (AMP) production.

## Materials and methods

### Mice

Wild-type C57BL/6 mice were bred and maintained under specific pathogen-free conditions in accredited animal facilities at the University of Oxford. Colitis was induced by infection with *H. hepaticus* and intraperitoneal injection of 1 mg 1B1.2 anti-IL10 receptor antibody (aIL10R) on days 0 and 7 (Kullberg et al., 2006; Izcue et al., 2008). Sixteen female pups were co-housed in four cages with four mice per cage from weaning onwards. From these four cages, two cages were randomly assigned to the *H. hepaticus* infection group and two to the uninfected group. Within the *H. hepaticus* infection group, all mice were infected with *H. hepaticus* and two mice per cage were injected with aIL10R antibody on days 0 and 7 to induce colitis. Within the uninfected group two mice per cage were injected with aIL10R antibody on days 0 and 7 and two mice per cage were left entirely untreated. Mice were fed irradiated standard chow (BK001E product code 801965) from Special Diets Services (www.sdsdiets.com). Mice were killed on day 14 of the experiment and the colon was removed for histology and collection of colon contents. All procedures involving animals were conducted according to the requirements and with the approval of the UK Home Office Animals (Scientific Procedures) Acts, 1986. Mice were negative for *Helicobacter* spp. and other known intestinal pathogens and were 10 weeks old when experiments were started.

### Histological assessment of intestinal inflammation

Proximal, mid- and distal colon pieces were fixed in 10% buffered formalin solution. Paraffin-embedded sections were cut and stained with haematoxylin and eosin and inflammation was assessed using a previously described scoring system (Izcue et al., 2008).

### Metagenomic and metatranscriptomic library preparation and sequencing

Colon contents were immediately frozen on dry ice. For simultaneous DNA and RNA extraction, pellets were dissolved in 500 μl extraction buffer (200 mm NaCl, 20 mm EDTA, 4 m guanidine thiocyanate and 1% (v/v) β-mercaptoethanol) and 210 μl 20% (w/v) SDS and lysed with a bead beater twice for 2 min using Lysing Matrix E tubes (MP Biomedicals, Santa Ana, CA, USA; procedure modified according to Turnbaugh et al., 2009, 2010; McNulty et al., 2011). Samples were centrifuged at 8000 *g* for 5 min and the supernatant was diluted with three volumes of lysing buffer (ZR-Duet DNA/RNA Miniprep kit, Zymo Research). DNA and RNA were further extracted using the ZR-Duet DNA/RNA Miniprep kit (Zymo Research, Irvine, CA, USA) according to the manufacturer’s instructions. DNA libraries were prepared using the PrepX ILM 32i DNA library kit (Wafergen Biosystems, Fremont, CA, USA) and sequencing was performed on the Illumina HiSeq 2500 (Illumina, San Diego, CA, USA) resulting in an average of 51.10 M (45.68–62.42 M) 150 bp paired-end reads per sample. Total RNA was depleted of ribosomal RNA using Epibio’s (Epicentre, Madison, WI, USA) universal bacteria ribo-zero kit, single version. Libraries were constructed using the NEBNext Ultra directional RNA library prep kit (New England Biolabs, Ipswich, MA, USA, E7420L). RNA sequencing was performed on the Illumina HiSeq 2000 (Illumina) resulting in an average of 45.38 M (17.75–84.46 M) 100 bp paired-end reads per sample.

### Read processing

Illumina adaptor sequences were removed using cutadapt (https://pypi.python.org/pypi/cutadapt/1.4.2) and overlapping paired reads were combined using Flash (version 1.2.6) (Magoč and Salzberg, 2011).

### Metagenomic and metatranscriptomic sequence analysis

Metagenomic and metatranscriptomic data sets were aligned to the mouse genome (mm10) using the Burrows-Wheeler aligner (BWA) (Li and Durbin, 2009; bwa mem –M –k 25 –t 12) to remove host sequences from microbial analysis. RNA sequences were further aligned to the SILVA database (www.arb-silva.de/fileadmin/siva_databases) using BWA (Li and Durbin, 2009; m –M –k 25 –t 12) to remove any remaining ribosomal RNA sequences. Consistent with successful depletion of rRNA in our metatranscriptomic samples, we observed an average 4.59% (range 2.34–10.65%) of 16S rRNA contaminating reads (Supplementary Table S1). For taxonomic profiling, metagenomic and metatranscriptomic sequences were aligned to the NCBI non-redundant (NR) protein database (ftp.ncbi.nlm.nih.gov/blast/db/FASTA/nr.gz) using DIAMOND (version 0.3.9; Buchfink et al., 2014; http://ab.inf.uni-tuebingen.de/software/diamond) with default parameters. The lowest common ancestor approach implemented in a pre-released version of MEGAN tools (http://ab.inf.uni-tuebingen.de/data/software/megan5/download/mtools.zip; Huson and Weber, 2013) was used to assign reads at the level of genus (lcamapper.sh –ms 50 –me 0.01 –tp 50). Diversity and rarefaction analyses were performed on genus-level counts using the vegan package in R3.1.0 (https://www.r-project.org/). Differential abundance of genera was performed using the metagenomeSeq (Paulson et al., 2013) package from R/Bioconductor (https://www.bioconductor.org/). For functional analysis, the integrated gene catalogue (IGC; Li et al., 2014) was used as a reference. Metagenomic and metatranscriptomic sequences were aligned to the IGC gene database using DIAMOND (version 0.3.9) with default parameters. Each aligned read was assigned a NOG identifier (using IGC annotated evolutionary genealogy of genes: non-supervised orthologous groups, eggNOG) based on the annotation of the ‘best hit’ (highest bit score). We validated the use of the ‘best hit’ approach by showing that for >91% of aligned reads, >50% of alternative hits matched the annotation of the ‘best hit’ (Supplementary Figure S1). This demonstrated that the ‘best hit’ was an appropriate representative in this setting. Principal components analysis (PCA) was performed at the level of genus and NOG using metagenomeSeq normalised abundances using the prcomp function in R3.1.0. Differential abundance analysis was performed using the metagenomeSeq package from R/Bioconductor on per NOG counts. NOGs were called as differentially abundant at a Benjamini-Hochberg adjusted *P*-value <0.05.

### Replication metatranscriptomic data set

To increase power to detect significant changes in genera transcript abundance in colitis and confirm changes in NOG transcription, we generated a replication metatranscriptomic data set. Eight wild-type C57BL/6 mice were maintained under specific pathogen-free conditions in accredited animal facilities at the University of Oxford that were distinct from those used in the previous experiment. Mice were housed in two cages (five in one and three in the other) and all mice were colonised with *H. hepaticus* and treated with aIL10R antibody as described for the previous experiment. Mice were fed irradiated standard chow (RM3-P product code 801700) from Special Diets Services (www.sdsdiets.com). Faecal pellets were collected from the mice on day 0 of the model (before infection and treatment) and on day 14 (after induction of colitis). RNA extraction and library preparation was performed as described for the previous experiment. Sequencing was performed on the Illumina HiSeq 2500 platform (Illumina), generating an average of 34.12 M (range 23.78 M–46.27 M) 150 bp paired-end reads per sample. Data processing and analysis was performed as described for the previous experiment. Mice were killed on day 28 of the model and histology scores were calculated using the same method described for the previous experiment.

### Gene set enrichment analysis (GSEA)

To determine whether genera found to be significantly differentially abundant in the replication metatranscriptomic data set were enriched in the initial data set, we performed GSEA (Subramanian et al., 2005). Briefly, genera in the initial data set were ranked according to fold change (increasing or decreasing in colitis versus steady state) and we used an implementation of the GSEA pre-ranked function (https://github.com/CGATOxford/proj029/blob/master/scripts/GSEAPreranked.py) to assess enrichment of significant genera at the top of that list. In total, 1000 permutations of genus rank were used to derive a null distribution of enrichment scores and this was used to determine significance. This provided a measure of the equivalency of genus changes across replicate metatranscriptomic data sets in the absence of strong significant changes observed in the initial data set.

### Defining colitis-responsive NOGs

Fold changes in abundance between steady state and *H. hepaticus*+aIL10R were used as input into a regression model using DNA log_2_(fold change) and RNA log_2_(fold change) as predictor and dependent variables, respectively (lm function in R3.1.0). NOGs that were identified as being significantly higher/lower in abundance using the previously described metagenomeSeq analysis (at the level of DNA or RNA) were defined as colitis-responsive if they laid outside of the 95% prediction intervals of the linear model.

### Microarray analysis of mouse colon

In a separate experiment to the sequencing experiment, expression profiles for whole colon across seven time points following *H. hepaticus* and aIL10R treatment (the same protocol as described for the sequencing experiment with the exception of continued weekly injections of aIL10R antibody) were obtained using Illumina MouseWG-6-V2 microarrays (*n*=4/5 at each time point). Probes were used for downstream processing if they were expressed significantly above background (detection *P*-value <0.05) in at least four samples. This resulted in the analysis of 24 065 probes (out of a total of 45 289). Array signal intensities were background adjusted, transformed using the variance-stabilising transformation and quantile normalised using Lumi (Du et al., 2008) from R/Bioconductor. Differential expression analysis was performed for each time-point contrast using the empirical Bayes method in LIMMA (Ritchie, 2015). Significance was defined as a Benjamini-Hochberg adjusted *P*-value <0.05. The union of significantly different probes was visualised in a heatmap and probes were clustered into distinct sets using *k*-means clustering (*k*=3) as implemented in R (kmeans function in R3.1.0). Enriched Gene Ontology (GO) biological processes were assessed for each gene cluster using a hypergeometric test and were called significantly enriched at a Benjamini-Hochberg adjusted *P*-value <0.05.

### Differential abundance of host genes in faecal samples

To assess the expression of host genes in faecal contents, we used RNA sequence alignments to the mouse genome. The gene counts (annotations from mm10 Ensembl 74) were produced using gtf2table (sum over exons per gene) from the Computational Genomics Analysis Toolkit (CGAT; Sims et al., 2014) and these were used as input to DESeq (Anders and Huber, 2010; version 1.17.0) from R/Bioconductor. Differentially expressed genes between steady state and *H. hepaticus*+aIL10R were identified at a significance level of Benjamini-Hochberg adjusted *P*-value <0.05.

### Cell-type enrichment analysis

Cell-type enrichment analysis for genes overrepresented in the faeces of colitic mice was performed using the CTen web application (Shoemaker et al., 2012). Cell types were considered significant at a Benjamini-Hochberg adjusted *P*-value <0.01.

## Results

### Microbial community composition and transcriptionally active members

**Figure 1:**
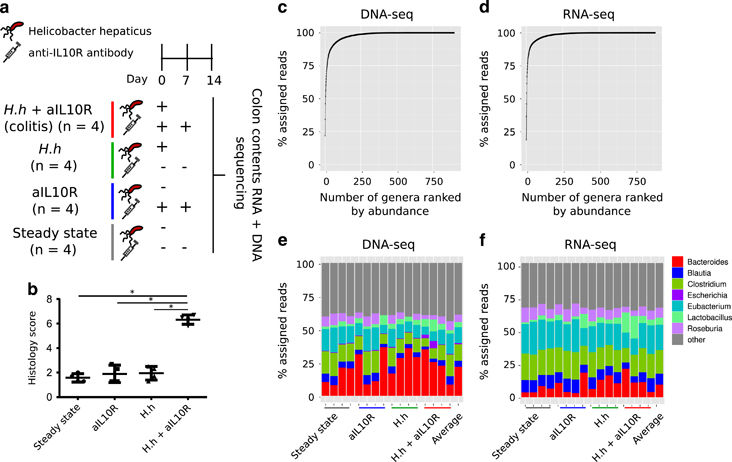
Microbial community composition across samples. (**a**) Overview of the experimental setup and number of mice in each group. (**b**) Histological assessment of mouse colon for mice in each group at day 14 of the model. (**c**) The average cumulative proportion of metagenomic reads assigned to genera. (**d**) The average cumulative proportion of metatranscriptomic reads assigned to genera. (**e**) The genera that constitute the majority of the metagenomic data (>5% assigned reads). (**f**) The genera that constitute the majority of the metatranscriptomic data (>5% assigned reads). H. h=H. hepaticus.

We sequenced RNA and DNA from gut contents of mice across four conditions (Figure 1a) that included a group of colitic mice treated with *H. hepaticus* and anti-IL10R antibody (aIL10R) and three different groups of control mice (Figure 1b). Reads were assigned to taxa using DIAMOND alignments followed by classification with the lowest common ancestor method. Sequence alignment statistics for both DNA and RNA data sets are provided in Supplementary Table S1. The metagenomic data set was dominated by sequences from Firmicutes (mean 56% (34–76%)) and Bacteroidetes (mean 29% (11–48%)) phyla, with additional contributions from Proteobacteria (mean 9% (7–15%)) and Actinobacteria (mean 3% (2–6%)). These four phyla thus contributed an average of 97% of sequences to our metagenomics data. Our metatranscriptomic data set was also dominated by Firmicutes (mean 78% (63–87%)) and Bacteroidetes (mean 12% (4–25%)), together contributing an average of 90% of sequences, with Proteobacteria and Actinobacteria contributing an average 6% (5–10%) and 2% (1–2%), respectively. These data confirm that the dominant phyla are active in the community and that the Firmicutes contribute the most to the community transcript pool. At the genus level, our metagenomic data set was composed of a few dominant genera along with many at lower abundance (Figure 1c), a feature that was consistent in our metatranscriptomic data set (Figure 1d). *Bacteroides* (mean 22% (8–37%)), *Clostridium* (mean 12% (6–18%)), *Eubacterium* (mean 12% (6–19%)), *Roseburia* (mean 6% (3–9%)), *Lactobacillus* (mean 4% (2–9%)), *Blautia* (mean 5% (2–7%)) and *Escherichia* (mean 1% (0.4–6%)) contributed >50% of assigned reads, with 896 low abundance (<5% assigned reads) genera making up the remainder (Figure 1e). We observed the same dominant genera contributing to our metatranscriptomic data set (Figure 1f). However, the rank order of abundance was different when compared with the metagenomic data set with *Eubacterium* (mean 19% (11–24%) and *Clostridium* (mean 18% (11–21%)) contributing the highest proportion of assigned reads, followed by *Bacteroides* (mean 10% (3–21%)), *Blautia* (mean 8% (5–10%)), *Roseburia* (mean 7% (4–10%)) and *Lactobacillus* (mean 5% (1–17%)). The remainder was made up of 881 low abundance (<5% assigned reads) genera (Figure 1e). Together, these data demonstrate that the dominant taxa in our data set are transcriptionally active although the rank order of genera based on DNA relative abundance is not completely recapitulated at the level of community transcription with *Eubacterium* and *Clostridium* being the dominant contributors to the mouse gut metatranscriptome.

### Alterations to community structure and transcriptional activity in colitis

**Figure 2:**
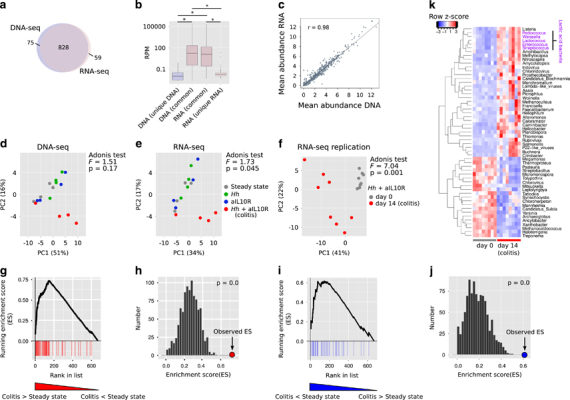
Differentially abundant genera in colitis versus steady state. (**a**) Overlap of genera detected with ⩾1 read in ⩾1 sample in metagenomic and metatranscriptomic analyses. (**b**) Distributions of reads per million (RPM) for genera detected in DNA-seq, RNA-seq or both data sets (average across 16 samples). *Wilcoxon rank-sum test *P*<0.05. (**c**) Correlation between genus abundance estimates (log2) of 669 genera detected at abundance >0.1 RPM in both data sets. (**d**) PCA of metagenomeSeq normalised genus abundances (669 genera detected at >0.1 RPM in both data sets) in metagenomic analysis. (**e**) PCA of metagenomeSeq normalised genus abundances (669 genera detected at >0.1 RPM in both data sets) in metatranscriptomic analysis. (**f**) PCA of metagenomeSeq normalised genus abundances (652 genera detected at >0.1 RPM in both RNA-seq data sets) in a replication metatranscriptomic data set. (**g**) Gene set enrichment analysis (GSEA) using the genera identified as significantly increased in abundance in the replication data set as the gene set for enrichment testing in the initial data set. The initial data set was ranked by fold change (high to low) in the comparison between colitis and steady state and tested for a significant enrichment at the top of the list using GSEA and permuted genus ranks with 1000 permutations as shown in (**h**). (**i**) GSEA of genera significantly decreased in colitis in the replication data set with the initial data ranked by fold change (low to high). Significance testing was again performed using 1000 permutations of genus rank as shown in (**j**). (**k**) Genera identified as being significantly differentially abundant in the replication metatranscriptomic data set and that contributed to the enrichment at the top of the ranked lists in (**g**) and (**i**) i.e. occurred in the ranked list before the maximum enrichment score was reached (the so-called ‘leading edge’ subset).

In the *H. hepaticus*+aIL10R model, colitis develops progressively from day 6, peaking at day 14 with high levels of cell infiltration, epithelial hyperplasia and goblet cell loss, presence of crypt abscesses and submucosal inflammation and a large area of affected tissue (Kullberg et al., 2006; Schiering et al., 2014; Arnold et al., 2015). As expected, these features of inflammation were significantly higher in *H. hepaticus*+aIL10R mice in the present study as compared to *H. hepaticus* alone, aIL10R alone and steady state control groups (Figure 1b) demonstrating colitis induction by day 14. Using our metagenomic and metatranscriptomic data sets, we sought to assess changes to microbial community composition and transcriptional activity in the model. Our analysis workflow is provided in Supplementary Figure S2. Using DNA analysis at the genus level, we found no differences in diversity or richness in mice with colitis (*H. hepaticus*+aIL10R) versus those without (Supplementary Figure S3). There was a strong overlap of detected genera between data sets (Figure 2a) with differences attributed to under-sampling of low abundance genera (Figure 2b). Confirming the dependency of transcriptional activity on DNA abundance, we observed a strong correlation (*r*=0.98) between DNA and RNA abundance for genera detected in both data sets (abundance >0.1 reads per million (RPM), *n*=669; Figure 2c).

To assess changes to community composition and transcriptional activity in colitis, we used only those genera that were present at >0.1 RPM in both RNA and DNA data sets (*n*=669). PCA revealed altered community structure and community transcriptional activity in colitis with three out of four colitic samples being clearly differentiated from non-colitic control samples (that is, steady state, *H. hepaticus* alone and aIL10R groups) with significant clustering by condition found using metatranscriptomics (Figures 2d and e; PERMANOVA Adonis test *F*=1.73, *P*=0.045). Although in our model, we introduced an exogenous bacterium (*H. hepaticus*), its presence or absence did not drive the clustering of colitic samples as mice infected with *H. hepaticus* alone (non-colitic) clustered with steady state and aIL10R treated control mice (Figures 2d and e). These data are consistent with observed low proportions of *H. hepaticus* read assignments in infected mice (Supplementary Figure S4) and reflects that the colon is not the primary site of *H. hepaticus* colonisation.

Differential abundance analysis using metagenomeSeq revealed few statistically significant (Benjamini-Hochberg adjusted *P*<0.05) changes in colitis with 0 and 3 genera being differentially abundant in metagenomic and metatranscriptomic analyses, respectively. *Wolinella*, *Erysipelothrix* and *Epulopiscium* significantly altered in overall transcript abundance in inflammation. As we had observed a community shift during colitis (Figures 2d and e), we reasoned that a lack of significant differences in individual taxa abundance was due to low statistical power, at least at the level of genus abundance. To address this, we performed a replication experiment whereby we sequenced faecal RNA from eight mice prior to infection with *H. hepaticus* and treatment with aIL10R (day 0) and following infection and treatment at the peak of colitis (day 14; alignment statistics are given in Supplementary Table S1). Histology scores for colitic animals in this experiment were comparable to the original experiment (mean=5.85, s.d.=1.40). Again we saw a significant shift in community composition (Figure 2f; PERMANOVA Adonis test *F*=7.04, *P*=0.001). We also observed significant changes (adjusted *P*-value <0.05 and fold change >2) in genus transcript abundance for 88 genera (48 up and 40 down) in colitis (Supplementary Table S2). Both increased and decreased genera were significantly enriched among those genera that had the greatest fold differences between steady state and colitis in the original data set (Figures 2g–j), confirming equivalent community shifts in transcription between the two data sets. A significant enrichment was also observed for increased genera in the DNA data set but not for decreased genera (Supplementary Figure S5). Consistent increases in genus transcript abundance were observed for multiple members of lactic acid bacteria (LAB) that included *Pediococcus*, *Weissella*, *Lactococcus*, *Enterococcus* and *Streptococcus* (Figure 2k).

These data support reproducible community shifts in LAB transcription during colitis that, at least in part, are due to changes in genus abundance during colitis.

### Encoded and transcribed functions of the gut microbiota

**Figure 3:**
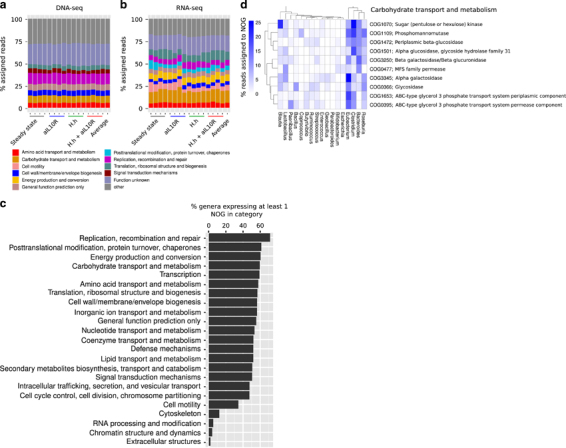
Functional profiling of the gut microbiota using metagenomics and metatranscriptomics. (**a**) The eggNOG functional categories that constitute the majority of the metagenomic data (>5% assigned reads). (**b**) The eggNOG functional categories that constitute the majority of the metatranscriptomic data (>5% assigned reads). (**c**) Metatranscriptomic reads were selected that could be assigned to both a genus and a NOG (average number of reads=6.3 M) and the proportion of genera that expressed any NOG in each of the eggNOG functional categories was plotted. (**d**) A breakdown of the genera that contributed to the transcription of the top 10 most highly abundant NOGs in the eggNOG functional category carbohydrate transport and metabolism. Only genera that contributed >1% of metatranscriptomic reads to any given NOG are shown.

By combining metagenomics and metatranscriptomics, we sought to compare the functional potential (that is, encoded functions) of the microbiota with transcriptional activity of those functions, as well as define the genera from which they derived. To this end, we aligned both DNA and RNA reads to the IGC and assigned them to orthologous genes (NOGs). We aligned an average of 32.64 M (68.3%) and 8.9 M (45.74%) of DNA and RNA pre-processed reads, respectively. These alignment statistics are in line with the maximum achieved in metatranscriptomic analyses to date (Ishii et al., 2013), validating our approach of using the IGC as the reference. At the level of eggNOG functional categories and excluding unknown and general functions, we observed that the highest proportion of metagenomic reads were assigned to replication, recombination and repair (mean 12% (11–13%)) followed by carbohydrate transport and metabolism (mean 8% (8–9%)), cell wall/membrane/envelope biogenesis (mean 6% (6–6%)), amino acid transport and metabolism (mean 6% (5–6%)), translation, ribosomal structure and biogenesis (mean 5% (4–5%)) and signal transduction mechanisms (mean 4% (3–6%); Figure 3a). Interestingly, we observed a different functional profile using metatranscriptomic data. The highest proportion of reads in these data were assigned to carbohydrate transport and metabolism (mean 14% (11–18%)), followed by translation, ribosomal structure and biogenesis (mean 9% (6–14%)), energy production and conversion (mean 8% (6–8%)), replication, recombination and repair (mean 6% (5–8%)), cell motility (mean 6% (3–11%)), amino acid transport and metabolism (mean 6% (5–7%)) and post-translational modification, protein turnover, chaperones (mean 5% (3–10%); Figure 3b). It is interesting to consider the observed differences between encoded and transcribed functions. The DNA abundance of any given function is determined by the number and abundance of taxa encoding that function. RNA abundance, however, is additionally determined by the importance of that function at the time of sampling. It is therefore unsurprising that replication, recombination and repair is the most abundant category at the DNA level as this category is ubiquitous among genera with >70% of genera expressing at least one NOG in this category (Figure 3c). The observation that this is not reflected in the RNA profile suggests that at least at the time of sampling, replication was not the dominant activity. Conversely, NOGs in the carbohydrate transport and metabolism category are widely expressed (~60% of genera express at least one NOG is this category) and are likely to be an important function of the community, particularly for those genera at high abundance. Indeed, among the most highly expressed NOGs are glycoside hydrolases (α- and β-galactosidases, α- and β-glucosidases) that are predominantly expressed from *Eubacterium*, *Clostridium*, *Bacteroides* and *Roseburia* (Figure 3d), which reflect the major function of these bacteria in the breakdown of complex dietary carbohydrates.

### NOGs increased in colitis are associated with adaptations to environmental stressors

**Figure 4:**
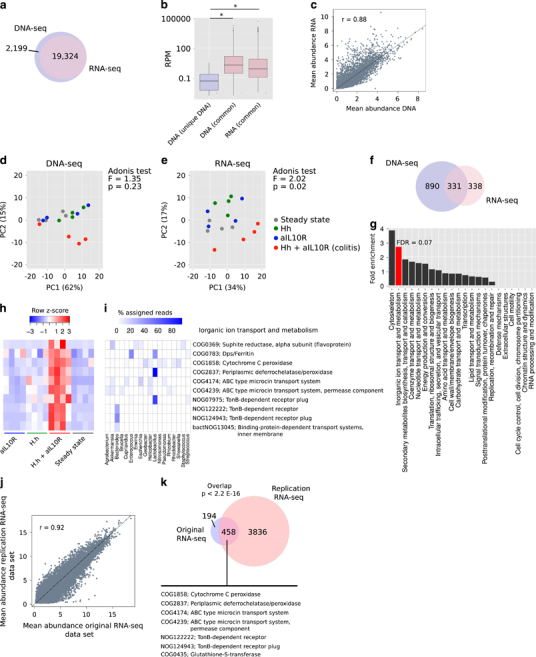
Identification of differentially abundant NOGs at the level of DNA and transcription. (**a**) Overlap of NOGs detected with ⩾1 read in ⩾1 sample in metagenomic and metatranscriptomic analyses. (**b**) Distributions of reads per million (RPM) for NOGs detected in DNA-seq, RNA-seq or both data sets (average across 16 samples). *Wilcoxon rank-sum test *P*<0.05. (**c**) Correlation between NOG abundance estimates (log2) of 14 211 NOGs that were detected at an abundance of >0.1 RPM in both data sets. (**d**) PCA of metagenomeSeq normalised NOG abundances (14 211 NOGs detected in both data sets at >0.1 RPM) in the metagenomic data set. (**e**) PCA of metagenomeSeq normalised NOG abundances (14 211 NOGs detected in both data sets at >0.1 RPM) in the metatranscriptomic data set. (**f**) Overlap of NOGs called as differentially abundant (adjusted *P*<0.05 in *H. h*+aIL10R (colitis) versus steady state) in metagenomic and metatranscriptomic data sets. (**g**) Fold enrichment of NOG functional categories in the set of NOGs that were found to be more highly abundant and transcribed in colitis versus steady state in both metagenomic and metatranscriptomic data sets. (**h**) Normalised RNA abundance of NOGs in the inorganic ion transport and metabolism eggNOG functional category that were found to be more highly abundant and transcribed in colitis versus steady state in both metagenomic and metatranscriptomic data sets. (**i**) The proportion of metatranscriptomic reads from each genus that contributed to transcription of each NOG identified in (**h**). Only those genera that contributed >1% of metatranscriptomic reads to any given NOG are shown. (**j**) Correlation of NOG abundance estimates (log2) across replicate metatranscriptomic data sets. The mean abundance across samples is plotted in each case. (**k**) Overlap of NOGs called as significantly differentially abundant (Benjamini-Hochberg adjusted *P*-value <0.05) across replicate metatranscriptomic data sets. The significance of the overlap was calculated using the hypergeometric test.

All NOGs identified in our metatranscriptomic data set were identified in the metagenomics data (Figure 4a). Those NOGs uniquely detected by DNA analysis were low abundance (Figure 4b) and therefore likely represented NOGs that were below the limit of detection in our metatranscriptomic data set. We further analysed those NOGs that were present at >0.1 RPM in both data sets (*n*=14 211) and observed a strong correlation of NOG abundances between data sets, although in contrast to genus-level abundances, NOG-level DNA abundance did not fully predict transcript abundance (Figure 4c). PCA of both data sets distinguished colitic samples from non-colitic control samples (Figures 4d and e), with clustering on NOG transcription providing the best separation of conditions (Figure 4e; PERMANOVA Adonis test; DNA *F*=1.35, *P*=0.23; RNA *F*=2.02, *P*=0.02).

Given the clustering together of control samples, we concentrated on differentially abundant NOGs between steady state and *H. hepaticus*+aIL10R conditions. Differential abundance analysis revealed 1221 and 669 significantly differentially abundant (metagenomics) and expressed (metatranscriptomics) NOGs in colitis, respectively (Figure 4f and Supplementary Table S3), and the overlap between the two data sets was highly significant (overlap=331, hypergeometric test *P*<0.01). The set of genes that were increased in colitis at the level of both DNA and RNA displayed a trend towards a significant enrichment for genes involved in inorganic ion transport and metabolism (Figure 4g; hypergeometric test FDR=0.07). Differentially abundant NOGs in this category were involved in multiple different processes (Figure 4h), including sulphite reduction to l-cysteine (COG0369), resistance to oxidative stress through either peroxidase activity (COG1858 and COG2837) or DNA protection (COG0783), microcin transport (COG4174 and COG4239) and nutrient transport (NOG09795, NOG124943 and bactNOG13045). Of interest, increases in cysteine (the precursor to glutathione) metabolic processes have been observed previously at the level of inferred function in human IBD and have been linked to the antioxidant properties of glutathione during inflammation (Morgan et al., 2012). Notably, in addition to an increase in abundance of cysteine biosynthesis from members of *Akkermansia*, *Escherichia*, *Erwinia* and *Staphylococcus* (Figure 4i), we also observed significant increases in DNA and transcript abundance of glutathionylspermidine synthase (COG0754) and glutathione-*S*-transferase (COG0435). These enzymes are key in maintaining the redox environment and regulating glutathione-dependent peroxidase activity in *Escherichia coli* during oxidative stress (Kanai et al., 2006; Chiang et al., 2010) and are consistent with increased glutathione metabolism in human IBD (Morgan et al., 2012). Further evidence for a role of oxidative stress on modulating the microbiota was the significant increase in DNA and transcript abundance of peroxidases (COG1858 and COG2837) from *Geobacter*, *Helicobacter*, *Nitrosomonas*, *Pseudomonas*, *Rhodobacter*, *Shewanella* and *Lactobacillus* as well as the DNA protection against starvation protein, *Dps/ferritin* (COG0783) from *Lactobacillus, Enterococcus* and *Streptococcus* (Figure 4i). We also observed significant increases in DNA and transcript abundance for ABC-type microcin transport systems (Figure 4i; COG4174 and COG4239). These orthologues constitute the *YejB* and *YejE* permease components of a transporter system that are expressed as part of the *yejABEF* operon in *Salmonella enterica serovar* Typhimurium and act to protect the bacterium from both bacteria- and host-derived AMPs (Eswarappa et al., 2008). Our data support the expression of these proteins across diverse bacteria (Figure 4i) and suggest a mechanism through which these bacteria are able to survive antimicrobial insult from other members of the microbiota and the host during inflammation. Previous reports have linked upregulation of microbial nutrient transport in human IBD (Morgan et al., 2012). We observed an increase in DNA and transcript abundance of components of the TonB-dependent receptors (Figure 4h)—protein complexes important in promoting active transport of rare nutrients including iron complexes (Schauer et al., 2008). The major contributors to the transcription of TonB-dependent receptor components in our data were *Lactobacillus* and *Bacteroides* (Figure 4i), suggesting that these genera are able to effectively compete for key nutrients in the inflamed environment. By using our replication metatranscriptomic data set, we were able to demonstrate reproducible estimates of NOG transcript abundance (Figure 4j; *r*=0.92) and confirm 458/652 (70%) of changes observed in the original metatranscriptomic data set that included the pathways described above (Figure 4k). A full list of differentially abundant NOGs identified in the replication metatranscriptomic data set is provided in Supplementary Table S4.

Together these data extend previous observations of increased metabolic processes related to oxidative stress and nutrient transport in human IBD by showing that these pathways are also changed at the level of transcription. We also reveal diverse mechanisms through which members of the microbiota may persist in the inflammatory environment.

### Colitis-responsive NOGs are involved in oxidative stress resistance

**Figure 5:**
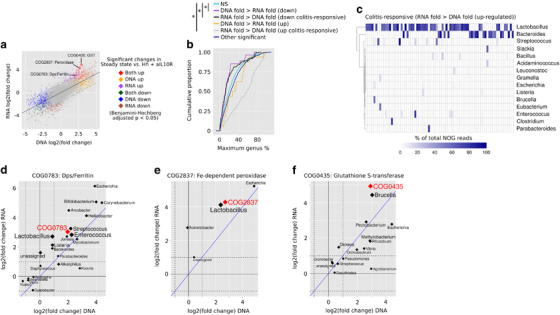
Transcription of upregulated, colitis-responsive NOGs is dominated by *Lactobacillus* and *Bacteroides*. (**a**) Correlation of fold changes between metagenomic and metatranscriptomic analyses. The solid line represents the linear model fit and dashed lines represent the 95% prediction intervals. NOGs lying outside of the 95% prediction intervals and that were called as differentially regulated in metatranscriptomic analysis were defined as colitis-responsive. (**b**) Metatranscriptomic reads were selected that mapped to both a genus and NOG (mean=6.3 M reads). The percentage of reads from each genus that contributed to NOG expression was calculated for all NOGs and the cumulative proportion was plotted for each NOG set. Significant differences in distributions were assessed using the Kolmogorov-Smirnov test for each set against the non-significant (NS) set of NOGs. (**c**) Heatmap displaying the percentage of metatranscriptomic reads (average across all samples) derived from each genus contributing to NOGs defined as being upregulated and colitis-responsive. Only those NOGs that have a major genus contributor (i.e., >50% contribution) are displayed. MetagenomeSeq was run on counts per genus per NOG and genera/NOG fold changes (log_2_) from metagenomic and metatranscriptomic analyses were plotted for genera that expressed (**d**) COG0783: Dps/ferritin (**e**) COG2837: Fe-dependent peroxidase and (**f**) COG0435: glutathione *S*-transferase. Points and text are scaled by relative RNA abundance. The blue solid line represents *y*=*x*. Solid black lines are where log_2_ (fold change) is 0 and dashed lines represent two fold changes.

Globally, changes in NOG DNA abundance predicted changes in NOG transcription (Figure 5a; linear model *R*^2^=0.57, *P*<2.2 × 10^−16^). However, we reasoned that a subset of NOGs that changed to a greater extent transcriptionally than would be predicted from changes in DNA abundance would be important in the microbial response to inflammation (colitis-responsive). Thus, we defined NOGs as colitis-responsive if they were significantly increased/decreased in colitis (DNA or RNA) and laid outside of the 95% prediction interval for a linear regression model using DNA log_2_(fold change) and RNA log_2_(fold change) as the predictor and dependent variables, respectively (Figure 5a). This analysis revealed 139 NOGs that were increased and colitis-responsive and a further 139 that were decreased and colitis-responsive. Strikingly, NOGs that were increased and colitis-responsive were more likely to be dominantly expressed from a given genus (Figure 5b), suggesting that certain taxa are more responsive to changing environmental conditions. Indeed, expression of these NOGs was dominated by *Lactobacillus* and *Bacteroides* (Figure 5c).

NOGs that we previously identified as being more highly abundant in colitis at both the DNA and transcript level that were annotated as *Dps/ferritin*, Fe-dependent peroxidase and glutathione *S*-transferase—genes involved in resistance to oxidative stress—were found to be increased and colitis-responsive (Figure 5a). The major contributing genera to changes in transcription of COG0783: *Dps/ferritin* were members of LAB (*Enterococcus*, *Lactobacillus* and *Streptococcus*), to COG2837: Fe-dependent peroxidase was *Lactobacillus* and to COG0435: glutathione *S*-transferase was *Brucella* (Figures 5d–f).

These data suggest that among functions that are at higher abundance in the colitic environment, those involved in oxidative stress resistance are the most responsive at the level of transcription.

### Relating microbial expression changes to host transcriptional responses in colitis

**Figure 6:**
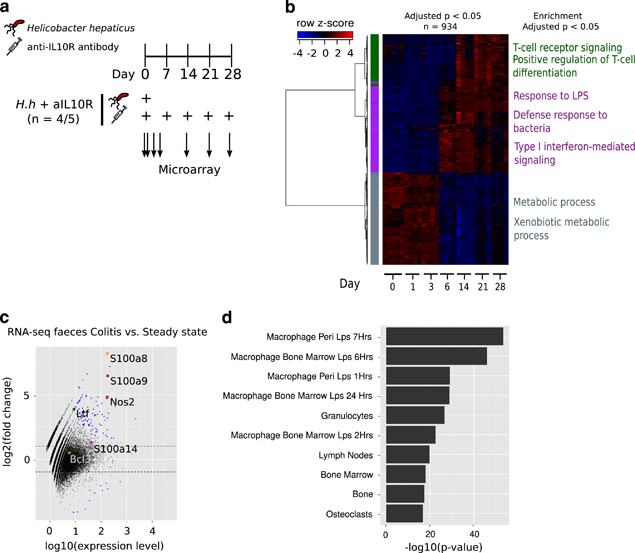
Luminal expression of host genes represents a signature of activated innate cells. (**a**) Experimental setup for characterising gene expression changes over the course of the colitis model. (**b**) Heatmap of colonic tissue transcriptional changes (LIMMA adjusted *P*<0.05) across time in the *H. h*+aIL10R model of colitis. Colours on the left panel represent cluster assignments for genes using *k*-means clustering (*k*=3). Gene Ontology (GO) biological processes that are significantly (Benjamini-Hochberg adjusted *P*<0.05) enriched for each cluster are labelled. (**c**) Differential expression analysis of mouse genes detected using RNA-seq from faecal samples between steady state and *H. h*+aIL10R (colitic) mice. Genes annotated as being involved in the antimicrobial defence response (GO biological process) are labelled. Dashed lines represent fold changes ⩽−2 and ⩾2. Blue=DESeq adjusted *P*<0.05. (**d**) Cell-type enrichment analysis of mouse genes identified as being more highly abundant in *H. h*+aIL10R (colitic) faeces compared to steady state. The top 10 enriched cell types are shown.

We aimed to determine inflammation-driven changes to host pathways that may contribute to the observed alterations to the microbiota. To this end, we profiled colonic tissue transcriptional responses from day 0 to day 28 in our colitis model using microarrays (Figure 6a). Colitic mice (day 14) in this experiment were of comparable severity to samples used for metagenomic and metatranscriptomic profiling (see Extended Data Figure 4a in Schiering et al., 2014). Over time we observed distinct patterns of mRNA regulation (Figure 6b and Supplementary Table S5). By using *k*-means clustering (*k*=3), we assigned each differentially regulated gene to a distinct cluster and performed pathway analyse of GO biological functions (all significantly enriched pathways are available in Supplementary Table S6). We found an enrichment of genes involved in innate immune responses as upregulated at the onset of colitis at day 6. These included multiple genes involved in bacterial defence, such as AMPs including *S100a8*, *S100a9*, *S100a14* and *Ltf* and nitric oxide synthesis (*Nos2*). To address the potential impact of host antimicrobial responses on the observed transcriptional changes in the microbiota, we next assessed the expression of host transcripts in the gut lumen using RNA-seq data from which the metatranscriptomic data were derived (Supplementary Table S1). Consistent with antimicrobial activity in the lumen of the gut during colitis, we observed significantly higher expression of the AMPs *S100a8*, *S100a9*, *S100a14* and *Ltf* as well as *Nos2* (Figure 6c) in colon contents. To predict the cell types that contributed to luminal transcripts, we used cell-type enrichment analysis among genes found to be increased in colitis. This analysis supported the presence of innate cells such as activated macrophages and granulocytes (Figure 6d) in the lumen during inflammation. An observed increased DNA and transcript levels of TonB-dependent receptor components and genes involved in resistance to oxidative stress from members of the microbiota suggest an adaptation of these microbes to withstand metal-ion sequestration through the formation of calprotectin from S100a8 and S100a9 and the oxidative stress response associated with activated macrophages and granulocytes in the lumen of the gut.

## Discussion

In this study, we have used shotgun metagenomics and metatranscriptomics to describe changes to community structure and function in colitis. In contrast to human IBD studies, we did not see a reduction in community diversity in mice with colitis although this is consistent with other mouse models of colitis—such as the DSS model (Berry et al., 2012). At the level of both genus and NOG abundances, we showed a high correlation of average abundance estimates between DNA and RNA data sets (*r*=0.97 and *r*=0.88, respectively), which is consistent with previous analyses in healthy human subjects (Franzosa et al., 2014) and reflects the predictive power of DNA abundance on RNA transcription. However, despite these high correlations, we have shown that metatranscriptomics at the level of NOG abundance is better able to discriminate between experimental conditions than DNA data sets, suggesting that metatranscriptomics provides information above and beyond that provided at the genomic level.

We observed significant community shifts at the level of genus transcription with significant increased transcript abundance from members of LAB, including *Pediococcus*, *Weissella*, *Lactococcus*, *Enterococcus* and *Streptococcus*. These findings are of note as LAB are generally considered to have anti-inflammatory properties through their ability to reduce the impact of oxidative stress on the host through superoxide dismutase and catalase activity (LeBlanc et al., 2011). Furthermore, increased abundance of *Enterococcus* and *Streptococcus* species have been reported in Crohn’s disease patients with recurring disease post surgery (De Cruz et al., 2015). Our data suggest that the LAB are well adapted to the inflamed environment although the influence of increased LAB transcription on exacerbation or resolution of disease remains to be determined.

Consistent with previously inferred microbial functional changes in human IBD, we observed higher abundance and transcription of genes involved in glutathione synthesis, metabolism and function in colitis (Morgan et al., 2012). An observed increase of sulphite reductase (COG0369), glutathionylspermidine synthase (COG0754) and glutathione *S*-transferase (COG0435) support a role for glutathione and glutathione conjugates (for example, glutathionylspermidine (Chiang et al., 2010)) in controlling oxidative damage during colitis. Further supporting a role for resistance to oxidative stress during colitis was the observed increase in abundance and expression of *Dps/ferritin* (COG0783) and Fe-dependent peroxidase (COG2837). These genes, along with glutathione *S*-transferase (GST), COG0435, were also defined as colitis-responsive, suggesting that they are transcriptionally induced in response to inflammation. In *E. coli*, *Dps/ferritin* provides resistance to oxidative stress through its ability to bind and protect DNA from oxidative damage (Wolf et al., 1999; Martinez and Kolter, 1997) and by reducing the production of hydroxyl radicals via its ferroxidase activity (Zhao et al., 2002) while Fe-dependent peroxidases are vital for the reduction of endogenously produced hydrogen peroxide by members of Lactobacilli (Martín and Suárez, 2010). The ability of Proteobacteria to withstand oxidative stress has been proposed as a mechanism through which they are able to expand in human IBD (Morgan et al., 2012; Haberman et al., 2014). Interestingly, while we observed a contribution from Proteobacteria—specifically *Brucella*—on transcriptional changes in glutathione-S-transferase (GST) (COG0435), changes in both *Dps/ferritin* and Fe-dependent peroxidase were driven by transcription from the LAB *Lactobacillus*, *Enterococcus* and *Streptococcus*. Our analyses have therefore extended previous observations to include the LAB as responders to an inflamed environment through the induction of oxidative stress-response pathways. These data also provide an explanation for the observed increases in LAB abundance—the LAB are able to expand in the inflamed niche.

Transcriptomic profiling of gut tissue revealed an expected induction of the innate immune response at the onset of colitis. Interestingly, a transcriptional signature of activated macrophages and granulocytes was also observed in the lumen of the gut, implicating a role for these cell types in altering the luminal niche during inflammation. Increased transcription of AMP transcripts, such as *S100a8*, *S100a9*, *S100a14* and lactoferrin (*Ltf*) in the lumen of the gut during colitis suggests a required ability for commensal microbes to withstand antimicrobial activity during inflammation. The mechanism of action of these antimicrobials is to sequester key nutrients, such as manganese and zinc and thus limit their availability to bacteria (Diaz-Ochoa et al., 2014). It is of particular interest therefore, that we observed an increase in DNA and transcript abundance of the TonB-dependent receptor complexes from members of the microbial community that would be predicted to facilitate the uptake of rare nutrients as a result of sequestration by host proteins.

Direct antimicrobial activity can occur through bacterial or host expression of antibacterial products that are capable of inducing bacterial cell death through membrane binding. An upregulation of bacterial ABC-type transporters related to the *yejABEF* operon of *Salmonella enterica serovar* Typhimurium that are required for resistance to both bacterial and host AMPs (Eswarappa et al., 2008) suggests an adaptation of community members to resist direct targeting of AMPs during inflammation.

Nutrient availability is a feature that determines metabolic function of the microbiota. Our microarray data revealed a downregulation of genes involved in xenobiotic and metabolic processes in colitic tissue at day 6 and included multiple enzymes involved in glycosylation of endogenous and exogenous substrates, including glucuronidation (*Ugt1a1*), sialic acid conjugation (*St6gal1*) and α(1,3) fucosylation (*Fut4*). Glycosylated proteins potentially act as substrates for a number of colonic microbes. Indeed, altered glycosylation during colitis and its impact on the microbiota is supported by recent evidence that IL22-induced *Fut2* expression on epithelial cells of the small intestine increases the expression of fucose-degrading enzymes in the microbiota—changes which contribute to host recovery (Pickard et al., 2014) and protection against invading pathogens (Pham et al., 2014; Pickard et al., 2014). It is therefore of interest that we also observed a downregulation of glycoside hydrolase family 30 (COG5520) enzymes in our microbiome experiment. Although speculative at present, this is of note as this family of enzymes have β-glucuronidase and β-fucosidase activity (Cantarel et al., 2009), which supports an hypothesis of transcriptional downregulation of these enzymes in response to downregulation of these moieties on host cells.

Both increased and decreased abundance of functions related to flagellar assembly have been reported previously in mouse models of colitis (Rooks et al., 2014; Schwab et al., 2014). In our data, we observed significant increased DNA abundance of three NOGs associated with flagellar assembly including a basal body-associated protein (NOG67631) and two flagellar L-ring proteins (COG1706 and COG2063). We did not observe any significant decreases in DNA or transcript abundance of NOGs annotated as being involved in flagellar assembly. Our results therefore support an increase in the capacity for flagellar assembly in colitis, a feature that is not supported at the level of transcription in our data.

Previous metatranscriptomic analyses in DSS-induced colitis (Berry et al., 2012; Schwab et al., 2014) suggest that *Lactobacillus*, *Enterococcus*, *Parabacteroides*, *Mucispirillum*, Lachnospiraceae and Enterobacteriaceae are indicator phylotypes of colitis (Berry et al., 2012). Together with our data, this suggests that changes in transcript abundance of the LAB including *Enterococcus* are consistent features across models of murine colitis.

## Conclusions

We have provided the first description of the transcriptional response of the gut microbiome in colitis using a combination of metagenomics and metatranscriptomics and have identified key functions that change in transcription in response to an altered gut niche. Specifically, increased abundance of NOGs involved in oxidative stress resistance, rare nutrient uptake and defence against AMPs from diverse members of the community suggest that these are genomic features that contribute to the success of these organisms in withstanding innate immune responses during inflammation.

## Supplementary information

Supplementary Figure 1 (PDF 59 kb)

Supplementary Figure 2 (PDF 33 kb)

Supplementary Figure 3 (PDF 31 kb)

Supplementary Figure 4 (PDF 57 kb)

Supplementary Figure 5 (PDF 117 kb)

Supplementary Table 1 (XLS 39 kb)

Supplementary Table 2 (XLS 30 kb)

Supplementary Table 3 (XLS 247 kb)

Supplementary Table 4 (XLS 375 kb)

Supplementary Table 5 (XLS 102 kb)

Supplementary Table 6 (XLS 25 kb)
