# Correction: Defining the microbial transcriptional response to colitis through integrated host and microbiome profiling

**DOI:** 10.1038/s41396-019-0539-0

**Published:** 2019-11-20

**Authors:** Nicholas Edward Ilott, Julia Bollrath, Camille Danne, Chris Schiering, Matthew Shale, Krista Adelmann, Thomas Krausgruber, Andreas Heger, David Sims, Fiona Powrie

**Affiliations:** 1 Kennedy Institute of Rheumatology, University of Oxford, Oxford, UK; 2 Institute for Tumor Biology and Experimental Therapy, Georg-Speyer-Haus, Frankfurt am Main, Germany; 3 The Francis Crick Institute, Mill Hill Laboratory, London, UK; 4 Translational Gastroenterology Unit, Experimental Medicine, Nuffield Department of Medicine, John Radcliffe Hospital, Oxford, UK; 5 CeMM Research Center for Molecular Medicine of the Austrian Academy of Sciences, Vienna, Austria; 6 Computational Genomics Analysis and Training (CGAT), MRC Functional Genomics Unit, Department of Physiology, Anatomy and Genetics, University of Oxford, Oxford, UK

**Correction to: The ISME Journal**

10.1038/ismej.2016.40

Since publication of the original paper the authors realised the following funding body was missing from the article’s Acknowledgements:

“FP and this work was also supported by the European Research Council (ERC, Advanced Grant Ares(2013)3687660)".

The authors apologise for any inconvenience caused.

