# Supplementary material for: Defining the microbial transcriptional response to colitis through integrated host and microbiome profiling
Source: ISME J. 2016 Mar 22;10(10):2389–404. doi: 10.1038/ismej.2016.40 (PMC5030693; doi:10.1038/ismej.2016.40)
Supplement: Supplementary file 1 — Supplementary Figure 1 (PDF 59 kb) [file 41396_2016_BFismej201640_MOESM249_ESM.pdf]

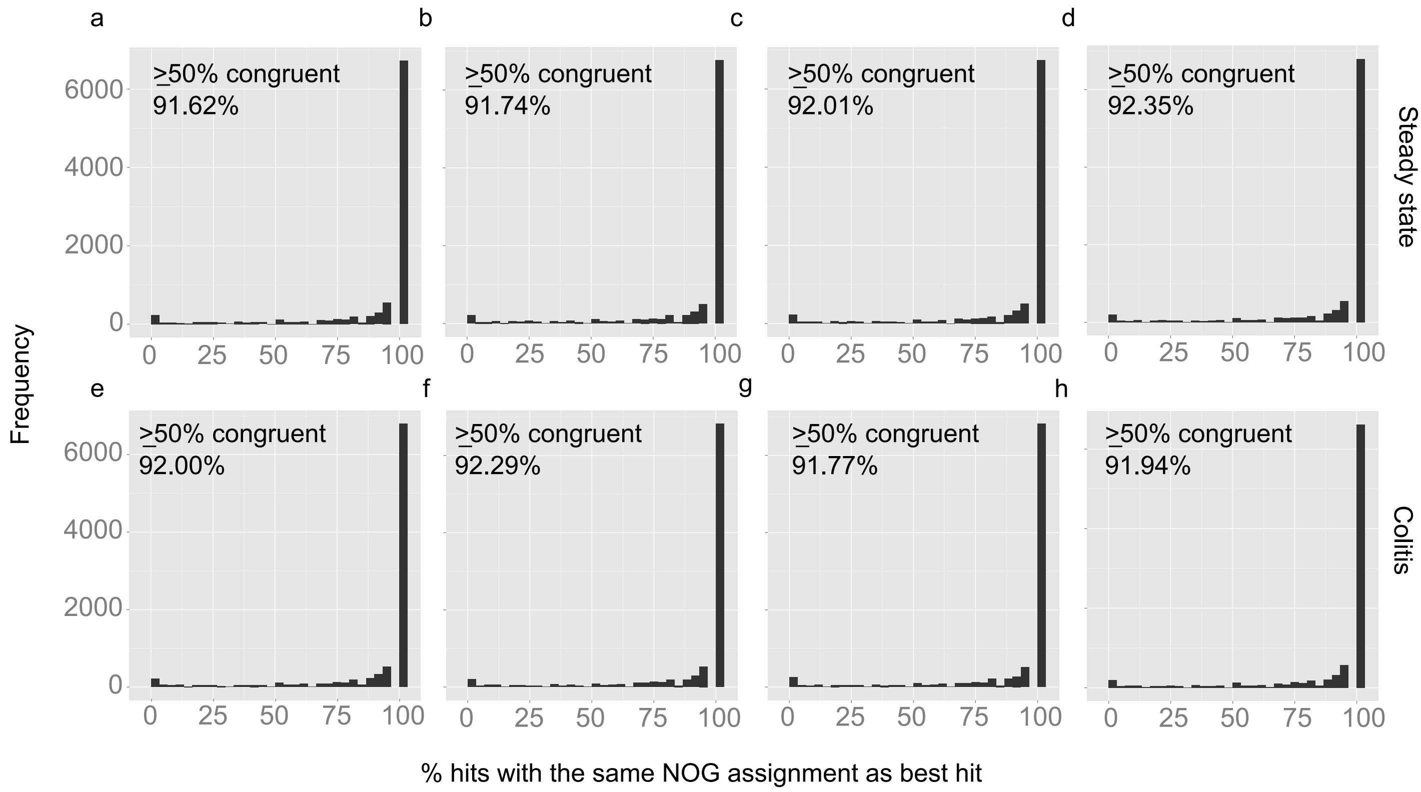

Supplementary Figure S1. DIAMOND best hit NOG is representative of additional hits. Histograms representing the distribution of percent congruency of alignment hits to the best hit for a sample of 10,000 aligned reads. In all cases, the majority of additional hits (>=50%) are congruent with the best hit in terms of NOG assignment.
