# Supplementary material for: Defining the microbial transcriptional response to colitis through integrated host and microbiome profiling
Source: ISME J. 2016 Mar 22;10(10):2389–404. doi: 10.1038/ismej.2016.40 (PMC5030693; doi:10.1038/ismej.2016.40)
Supplement: Supplementary file 2 — Supplementary Figure 2 (PDF 33 kb) [file 41396_2016_BFismej201640_MOESM250_ESM.pdf]

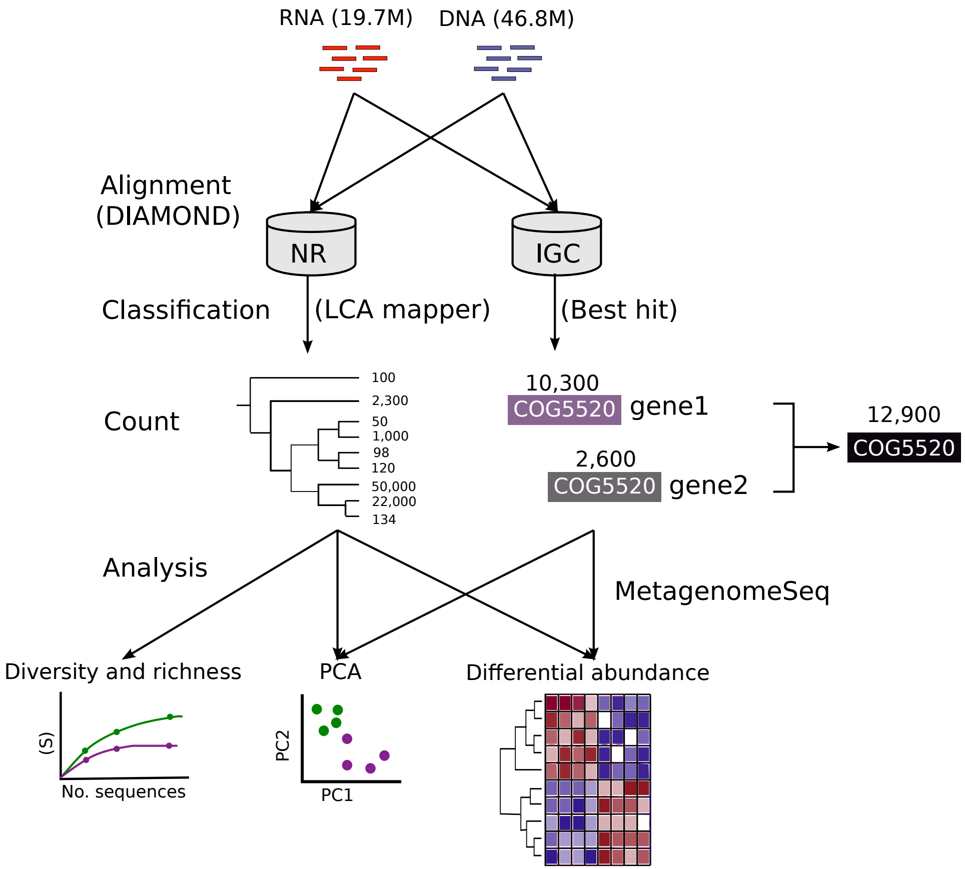

Supplementary Figure S2 Analysis workflow for taxonomic profiling and functional analysis of metagenomic and metatranscriptomic sequence data. NR = NCBI non-redundant protein database. IGC = Integrated Gene Catalogue.
