# Supplementary material for: Defining the microbial transcriptional response to colitis through integrated host and microbiome profiling
Source: ISME J. 2016 Mar 22;10(10):2389–404. doi: 10.1038/ismej.2016.40 (PMC5030693; doi:10.1038/ismej.2016.40)
Supplement: Supplementary file 3 — Supplementary Figure 3 (PDF 31 kb) [file 41396_2016_BFismej201640_MOESM251_ESM.pdf]

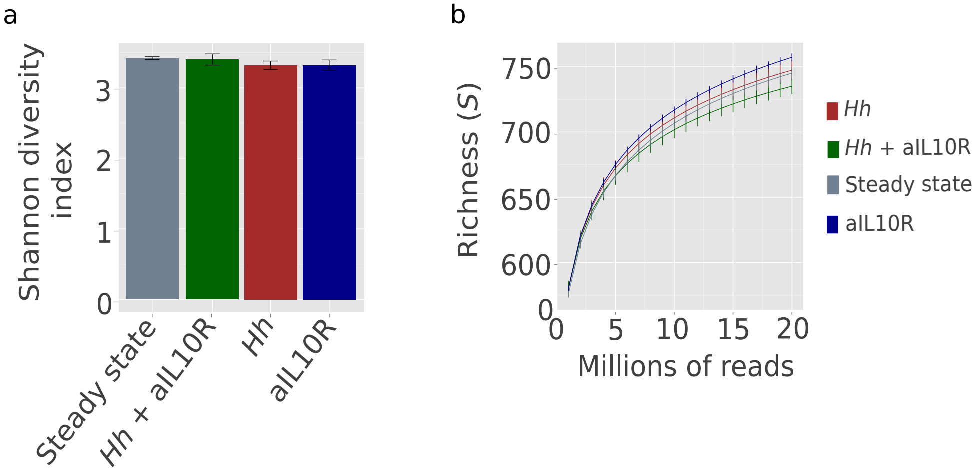

Supplementary Figure S3. Diversity and richness analysis of the gut metagenome. (**a**) Shannon diversity index as estimated using the Vegan package in R3.10. Bars represent the mean +/- se (**b**)Rarefaction analysis using the Vegan package in R3.10. At each sequencing depth we plot the mean +/- se for each condition.
