# Supplementary material for: Defining the microbial transcriptional response to colitis through integrated host and microbiome profiling
Source: ISME J. 2016 Mar 22;10(10):2389–404. doi: 10.1038/ismej.2016.40 (PMC5030693; doi:10.1038/ismej.2016.40)
Supplement: Supplementary file 4 — Supplementary Figure 4 (PDF 57 kb) [file 41396_2016_BFismej201640_MOESM252_ESM.pdf]

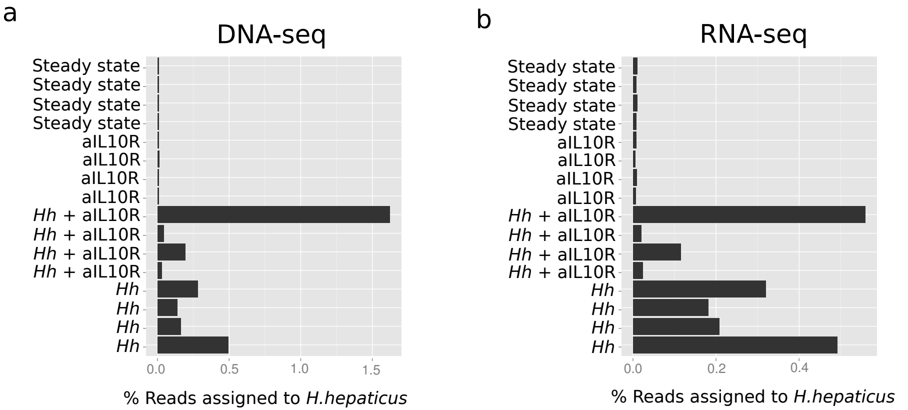

Supplementary Figure S4 Proportion of alignments assigned to Helicobacter hepaticus using the lowest common ancestor approach for (**a**) metagenomic and (**b**) metatranscriptomic data.
