# Supplementary material for: Defining the microbial transcriptional response to colitis through integrated host and microbiome profiling
Source: ISME J. 2016 Mar 22;10(10):2389–404. doi: 10.1038/ismej.2016.40 (PMC5030693; doi:10.1038/ismej.2016.40)
Supplement: Supplementary file 5 — Supplementary Figure 5 (PDF 117 kb) [file 41396_2016_BFismej201640_MOESM253_ESM.pdf]

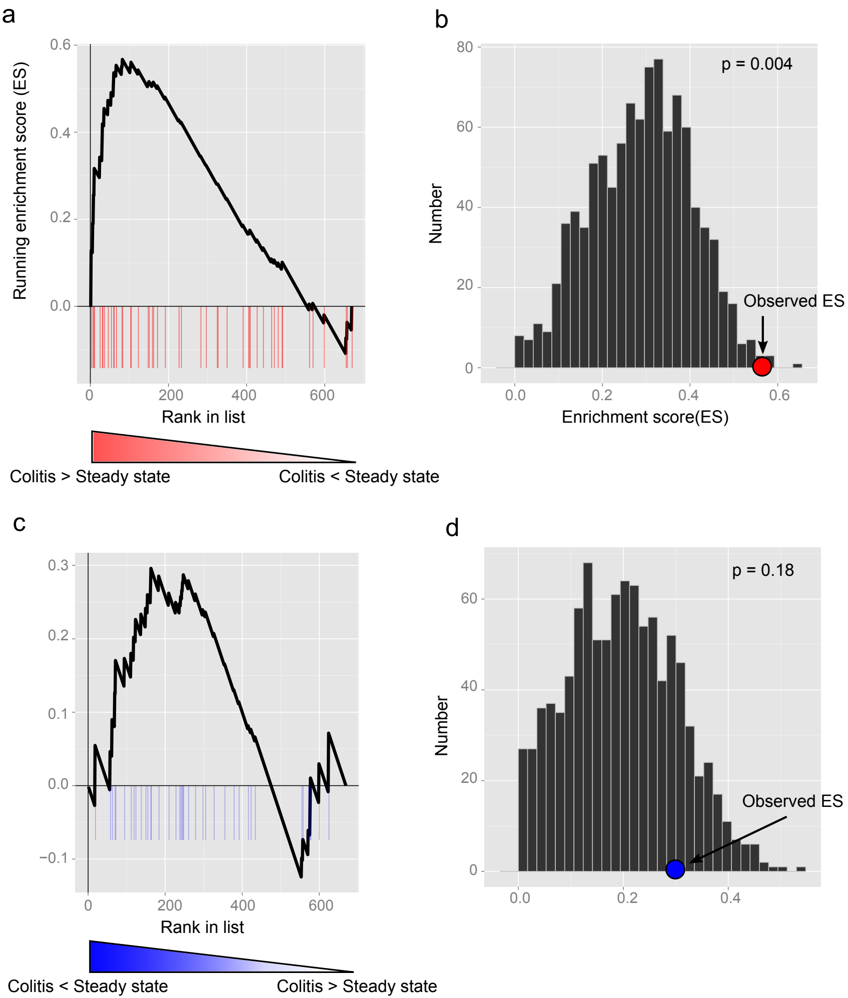

a
Running enrichment score (ES)
0.6
0.4
0.2
0.0
0
200
400
600
Rank in list
Colitis > Steady state
Colitis < Steady state
b
80
60
40
20
0
Number
p = 0.004
Observed ES
0.0
0.2
0.4
0.6
Enrichment score(ES)
c
0.3
0.2
0.1
0.0
−0.1
0
200
400
600
Rank in list
Colitis < Steady state
Colitis > Steady state
d
60
40
20
0
Number
p = 0.18
Observed ES
0.0
0.2
0.4
